# Carcass and meat traits of goats fed diets containing cottonseed cake

**DOI:** 10.5194/aab-64-395-2021

**Published:** 2021-09-20

**Authors:** Dallyson Yehudi Coura de Assis, Fabiano Almeida de Oliveira, Edson Mauro Santos, Ana Alice Lima de Gouvêa, Bruna Maria Aparecida de Carvalho, Camila de Oliveira Nascimento, Luís Gabriel Alves Cirne, Douglas dos Santos Pina, Aureliano José Vieira Pires, Henry Daniel Ruiz Alba, Gleidson Giordano Pinto de Carvalho

**Affiliations:** 1 Department of Animal Science, Universidade Federal da Bahia, Salvador, Bahia, Code 40170-110, Brazil; 2 Department of Animal Science, Universidade Federal da Paraíba, Areia, Paraíba, Code 58397-000, Brazil; 3 Institute of Agrarian Sciences, Universidade Federal de Minas Gerais, Montes Claros, Minas Gerais, Code 39404-547, Brazil; 4 Institute of Biodiversity and Forestry, Universidade Federal do Oeste do Pará, Santarém, Pará, Code 68035-110, Brazil; 5 Department of Animal Science, Universidade Estadual do Sudoeste da Bahia, Itapetinga, Bahia, Code 45700-000, Brazil

## Abstract

The cottonseed cake has the necessary nutritional characteristics to be able to substitute the traditional ingredients (such as soybean meal) and reduce the costs of the diet. However, it is necessary to determine the best level of inclusion of cottonseed cake in the diets of fattening goats to improve meat production and quality. The objective of this study was to evaluate carcass and meat traits of feedlot goats fed diets containing cottonseed cake replacing soybean meal (33 %, 66 % and 100 %). Thirty-two uncastrated Boer crossbred goats (4 months old, 16 ± 2 kg initial body weight) were used in a completely randomized experimental design. Replacing soybean meal with cottonseed did not compromise (P>0.05) slaughter weight, carcass traits (dressing percentage, loin-eye area and back-fat thickness), primal cuts or carcass morphometric measurements; moisture, protein, or total lipid contents of meat; or the physicochemical traits of color ($L^{*}$, $a^{*}$ and $b^{*}$ coordinates), pH, shear force, and cooking loss. However, there was a reduction (P=0.001) in the mineral matter content (from 1.08 % to 0.97 %) and an increase (P=0.006) in the cholesterol content (from 50.85 to 70.55 mg/100 g of meat) of the meat as the dietary levels of cottonseed cake were increased. Based on the results of production and meat quality, we recommend using cottonseed cake as an alternative protein source to replace up to 100 % of soybean meal in feedlot goat diets.

## 1 Introduction

Goats are hardy, prolific animals with high ability to adapt to adverse climatic conditions. The species is widespread around the world (Dubeuf, 2011; Iñiguez, 2011) and constitutes an important source of meat, milk, and skin. However, when reared in extensive systems in the semiarid environment, which is characterized by seasonal herbage production, their production performance is not satisfactory.

For these animals, feedlotting can be used as a tool to improve weight gain, carcass quality, and the meat product, as a result of a younger age at slaughter. To achieve these ends, goat diets must include traditional feedstuffs such as soybean and corn, which can account up to 70 % of the costs of an animal production system (McGrath et al., 2018).

Therefore, producers seek alternative feed sources that allow a reduction in production costs and minimize nutritional deficiency for the development of meat goat farming. In this context, the use of biodiesel by-products in the diet of these animals may be a viable alternative, given their positive effects on meat and milk quality, as observed in other animal species (Gonzaga Neto et al., 2015; Oliveira et al., 2015). Cotton by-products have nutritional characteristics that render them good sources of nutrients for animal feeding. Cottonseed cake, for instance, has satisfactory protein (> 30 %) and gross energy (> 5.5 kcal/g per dry matter) contents for use in ruminant diets (Rekwot, 2004).

Pereira et al. (2016) evaluated the use of cottonseed cake in the diet of feedlot lambs and found that it improved the nutritional properties of their meat. However, despite this potential, there is still a dearth of research investigating this cotton by-product in finishing goat diets. On this basis, the present study proposes to examine the effects of replacing soybean meal with cottonseed cake on carcass and meat traits of feedlot goats.

## 2 Material and methods

### 2.1 Experimental area

The experiment was conducted at the Experimental Farm at the School of Veterinary Medicine and Animal Science at the Federal University of Bahia (EMEVZ-UFBA), located in São Gonçalo dos Campos – BA, Brazil (12${}^{\circ }$23${}^{\prime }$57.51${}^{\prime \prime }$ S and 38${}^{\circ }$52${}^{\prime }$44.66${}^{\prime \prime }$ W). All experimental procedures were in compliance with the Brazilian law on research involving animals and were approved by the Ethics Committee on Animal Use of EMEVZ-UFBA (approval no. 08/2013).

### 2.2 Animals, facilities, and feeding management

Thirty-two Boer crossbred goats (4 months old, 16 ± 2 kg initial body weight) were used in the study. The animals were housed in individual suspended, slatted-floor pens (approximately 1.0 m${}^{2})$ equipped with individual feeding troughs and drinkers, inside a covered shed. At the start of the experiments, the goats were tagged, dewormed against ectoparasites, immunized with a multipurpose vaccine against clostridial infections, supplemented intramuscularly with vitamins A, D, and E, and allotted at random to the treatments, in a completely randomized experimental design.

The experimental period was 84 d, which were preceded by 12 d of adaptation to the environment, management and diets. During this period, the animals were fed Tifton 85 grass (*Cynodon* sp.) hay and increasing amounts of a concentrate containing cottonseed cake, as follows: 80:20 (roughage-to-concentrate ratio; day 1), 70:30 (day 4), 60:40 (day 7), and 50:50 (day 10). On the first evaluation day (day 13), the goats received a total diet with 50 % roughage and 50 % concentrate, which was maintained throughout the experiment.

The diets were provided in two daily meals – 50 % at 08:00 and 50 % at 16:00 – allowing 10 % orts. Tifton 85 grass hay, which was used as a roughage source, was previously ground to particles of approximately 2 cm. The concentrate portion of the diet was composed of ground corn, soybean meal, cottonseed cake, urea, ammonium sulfate, and a mineral supplement.

Treatments were represented by total diets in which soybean meal was replaced with cottonseed cake at 330, 660, or 1000 g/kg per dry matter (Table 1). The diets were formulated in accordance with the recommendations of the National Research Council (NRC, 2007), aiming to meet the requirements of goats with an estimated average daily gain of 150 g.

**Table 1:** Centesimal composition of ingredients and chemical composition of experimental diets.

Composition (g/kg per dry matter)	0	330	660	1000
Cottonseed replacement level (g/kg per dry matter)				
Tifton-85 hay	500	500	500	500
Ground corn	349	347	345	343
Soybean meal	120	80	40	0
Cottonseed cake	0	40	80	120
Urea + ammonium sulfate	16	18	20	22
Mineral mixture${}^{*}$	15	15	15	15
Chemical composition (g/kg per dry matter)				
Dry matter	909.8	902.2	910.3	910.6
Mineral matter	5.73	5.74	5.74	5.74
Crude protein	147.7	141.7	141.2	143.4
Ether extract	23.2	28.6	32.5	36.8
NDFap	386.7	400.5	414.4	428.2
Acid detergent fiber	212.5	221.0	229.4	237.9
Lignin	31.6	35.9	40.2	44.5
Total carbohydrates	755.7	754.3	753.0	751.6
Non-fibrous carbohydrates	369.0	353.8	338.6	323.4
Total digestible nutrients	684.3	677.5	670.6	663.8

${}^{*}$ Provides per kilogram, in active ingredients: calcium 120.00 g; phosphorus 87.00 g; sodium 147.00 g; sulfur 18.00 g; copper 590.00 mg; cobalt 40.00 mg; chromium 20.00 mg; iron 1800.00 mg; iodine 80.00 mg; manganese 1300.00 mg; selenium, 15.00 mg; zinc 3,800.00 mg; molybdenum 300.00 mg; and maximum fluorine 870.00 mg. phosphorus (P) solubility in citric acid (min. 2 %) – 95 %.

Samples of diet ingredients and orts were harvested once weekly throughout the experimental period. These were packed in labeled plastic bags which were then stored in a freezer at -20 ${}^{\circ }$C until analysis. After thawing, the samples were pre-dried in a forced-air oven at 55 ${}^{\circ }$C for 72 h and then ground through Willey knife mills to 1 mm particles which were subsequently stored for later laboratory analysis.

The chemical composition of the samples was analyzed in accordance with the analytical procedures suggested by the Association of Analytical Communities (AOAC, 1990), whereby the dry matter (DM; method 967.03), mineral matter (MM; method 942.05), crude protein (CP; method 981.10), and ether extract (EE; method 920.29) contents were determined. Neutral (NDF) and acid (ADF) detergent fiber contents were determined by the methodology proposed by Van Soest et al. (1991). The NDF content was corrected for the residual ash and protein (NDFap); the NDF residue was incinerated at 600 ${}^{\circ }$C for 4 h to correct the ash, while protein was corrected by subtracting the protein content in the NDF residue. Lignin was determined by method 973.18 of AOAC (2002), using the ADF residue. Non-fibrous carbohydrate (NFC) contents were calculated by the following equation, proposed by Detmann and Valadares Filho (2010): NFC = 100 - (% MM - % EE - % NDFap - % CP). Lastly, the total digestible nutrients (TDNs) were estimated by the formula suggested by Weiss (1999): TDN = DCP + 2.25 × DEE + DNFC + DNDF, where DCP, DEE, DNFC, and DNDF are the digestible fractions of CP, EE, NFC, and NDF, respectively.

Orts were harvested daily before the morning feed supply and weighed on a digital scale to determine DM intake, which was calculated as the difference between the amounts of the nutrient present in the diet and in the orts, as given by the following formula: intake (kg) = nutrient intake - nutrient in orts.

### 2.3 Slaughter

On the 84th day in the feedlot, the animals were transferred to a commercial meat-packing plant in the municipality of Pintadas (BA, Brazil). Once in the abattoir, the goats were fasted for 16 h, during which period they remained at rest. Subsequently, they were weighed to determine the post-fasting slaughter weight (FSW).

The animals were stunned by electronarcosis, which was followed by the bleeding, skinning, and evisceration steps, in accordance with the current procedures provided for by the guidelines for humane handling, transport, and slaughter of livestock (FAO, 2001). The gastrointestinal tract content (measured as the difference between full and empty gastrointestinal tract) was used to determine empty body weight (EBW = FSW - gastrointestinal content), as proposed by Resende et al. (2017).

### 2.4 Carcass traits, morphometric measurements, and primal cuts

Once eviscerated, the carcasses were weighed to determine hot carcass weight (HCW), which was then used to determine hot carcass yield (HCY = HCW / FSW × 100). Next, the carcasses were transferred to a cold chamber at a temperature of 6 ${}^{\circ }$C, where they remained 24 h hung by the tendons of the gastrocnemius muscle on appropriate hooks. Subsequently, the dressing percentage was calculated as the ratio between HCW and EBW.

Loin-eye area (LEA) was determined by making a transverse section between the 12th and 13th thoracic vertebrae and outlining the area corresponding to the cranial portion of the loin on a transparency sheet. The following measurements were thus made: length (A) and maximum depth (B) of the longissimus lumborum muscle (in cm), which were obtained using a ruler. Loin-eye area was then calculated by the following ellipse formula proposed by Silva Sobrinho (2003): LEA = (A/2*B/2) π (in cm${}^{2}$). Back-fat thickness (BFT) was measured in millimeters, using a digital caliper, at a 3/4 distance from the medial side of the longissimus muscle towards the spinous process.

After these analyses, the following carcass morphometric measurements were made: internal and external carcass length, chest and rump widths, chest depth, rump and thigh circumferences, and leg length. The length and circumference measurements were taken using a tape measure, whereas the width and depth measurements were obtained using a manual horse measuring stick.

The carcasses were divided lengthwise into two halves. The left half was sectioned into five anatomical regions called “primal cuts”, namely, neck, shoulder, ribs, loin, and leg. The right and left loins from each animal were packed, labeled, and frozen (-20 ${}^{\circ }$C) for later analyses of color, cooking loss (CL), shear force (SF), and centesimal composition.

### 2.5 Evaluation of meat physicochemical traits

After slaughter, the pH was measured in triplicate in the longissimus lumborum muscle, between the 12th and 13th thoracic vertebrae, using a stick-type digital pH meter (HI 99163m HANNA) with a penetration electrode.

The loins were thawed inside the plastic bags in a bio-oxygen demand (BOD) incubator at 10 ${}^{\circ }$C for 12 h and then dissected with a scalpel and a knife. Subsequently, the color was determined in the right loins using a colorimeter (Minolta CR-400) based on the CIELAB color space ($L^{*}$ (lightness), $a^{*}$ (red intensity) and $b^{*}$ (yellow intensity)) calibrated to a white standard. Muscle color was determined in the inner part of the muscle 5 min after the cut was made in order to expose the myoglobin to oxygen, following Miltenburg et al. (1992).

To determine CL, the samples were weighed and cooked in an industrial oven pre-heated to 170 ${}^{\circ }$C until the internal temperature in the geometric center of the samples reached 71 ${}^{\circ }$C, which was measured with a thermocouple with a digital reader. Upon reaching that temperature, the samples were removed from the oven and weighed again for the calculation, with results expressed in percentage terms. Next, to determine meat tenderness based on SF (expressed in N/cm), the cooked samples were cut into 25×25 mm cubes, in triplicate, and sectioned in the transverse direction of the muscle fibers using a texture analyzer (“texturometer”) with a Warner-Bratzler blade, following the methodology of Wheeler et al. (1995).

For the chemical composition analysis, the samples were freeze-dried for 72 h and then ground through a ball mill to generate the laboratory sample. The chemical composition of the meat was determined by measuring the moisture, MM, CP, and total lipid contents, following the methodology proposed by AOAC (1990).

Cholesterol was quantified by the enzymatic methodology, using Laborlab S/A laboratory kits, which were composed of two color reagents (no. 1, containing 0.025 mol/L of 4 aminophenazone and no. 2, containing 0.055 mol/L of phenol), in addition to an enzyme reagent (cholesterol oxidase 3 U/mol, POD 20 U/mol, lipase 300 U/mol). The working reagent was prepared by adding 0.5 mL of color reagent no. 1, 0.5 mL of color reagent no. 2, 19 mL distilled water, and 0.4 mL of the enzyme reagent. After adding 3 mL of the working reagent to the samples, a thermal treatment was carried out for 10 min at 37 ${}^{\circ }$C in a water bath. After a 90 min rest, absorbance was read against the blank, also prepared at 499 nm. The calibration curve was constructed from a standard cholesterol solution (1006 mg/100 mL) of concentration ranging from 0.01 to 0.05 mg/mL.

### 2.6 Statistical analysis

The experiment was set up as a completely randomized design with four treatments and eight replicates, totaling 32 experimental units. Results were evaluated by variance and regression analyses, with the degrees of freedom decomposed by polynomial orthogonal contrasts into linear or quadratic effects, according to the cottonseed cake levels. The significance of regressions was determined by the F test at the 5 % probability level of type-1 error, using the PROC GLM and REG procedures of Statistical Analysis System (SAS, 2009). P values between 0.05 and 0.10 were considered as trends.

## 3 Results

The intakes of DM, CP, NDF, NFC, and TDN were not changed (P > 0.05) by the dietary levels of cottonseed cake replacing soybean meal (Table 2). A quadratic effect on EE intake (P=0.013) and a quadratic trend on PC intake (P=0.079) were observed. Furthermore, the NFC intake showed a decreasing (P=0.056) linear trend.

**Table 2:** Intake of nutritional components (kg in the entire experimental period) of feedlot goats fed diets containing cottonseed cake replacing soybean meal.

Intake (kg)	Cottonseed cake level				SEM${}^{*}$	P value	
	(g/kg per dry matter)						
	0	330	660	1000		Linear	Quadratic
Dry matter	56.62	58.27	53.93	55.33	1.970	0.646	0.983
Crude protein	9.40	12.43	11.16	11.33	0.390	0.208	0.079
Ether extract${}^{*}$	0.93	0.73	0.85	1.00	0.019	0.297	0.013
NDFap	20.11	25.07	20.01	23.62	1.063	0.567	0.764
Non-fibrous carbohydrates	18.87	21.86	16.29	16.82	0.657	0.056	0.377
Total digestible nutrients	31.36	31.55	27.82	30.18	1.967	0.684	0.779

${}^{*}$ Standard error of the mean. $Y=0.921337-0.000717x+0.00000081x^{2}$, $R^{2}=0.88$. NDFap, neutral detergent fiber corrected for the residual ash and protein.

Post-fasting slaughter weight (average 22.47 kg), EBW (15.56 kg), HCW (12.00 kg), HCY (53.66 %), LEA (10.68 cm${}^{2})$, BFT (1.21 mm), and the yields of neck (6.93 %), shoulder (26.69 %), ribs (20.48 %), loin (11.73 %), and leg (34.17 %) also were not influenced (P>0.05) by the replacement of cottonseed cake with soybean meal in the diet (Table 3). However, neck (P=0.084) and leg (P=0.086) yield showed a quadratic trend.

**Table 3:** Quantitative traits and yields of primal cuts from the carcass of feedlot goats fed diets containing cottonseed cake replacing soybean meal.

Item	Cottonseed cake level				SEM${}^{*}$	P value	
	(g/kg per dry matter)						
	0	330	660	1000		Linear	Quadratic
Quantitative trait							
Post-fasting slaughter weight, kg	22.40	23.57	21.96	21.93	0.740	0.648	0.714
Empty body weight, kg	15.50	16.69	14.87	15.16	0.656	0.627	0.762
Hot carcass weight, kg	12.22	12.83	11.62	11.32	0.412	0.354	0.648
Hot carcass yield, %	54.80	54.34	53.32	52.16	0.611	0.116	0.784
Loin eye area, cm${}^{2}$	10.79	10.97	10.63	10.31	0.150	0.187	0.419
Back-fat thickness, mm	1.21	1.21	1.19	1.22	0.060	0.814	0.218
Cut yield (%)							
Neck	6.47	7.36	7.18	6.72	0.154	0.731	0.084
Shoulder	26.99	26.81	26.59	26.36	0.190	0.457	0.733
Ribs	19.76	21.17	19.73	21.26	0.354	0.354	0.769
Loin	12.35	11.23	11.89	11.45	0.373	0.682	0.654
Leg	34.43	33.43	34.61	34.21	0.283	0.504	0.086

${}^{*}$ Standard error of the mean.

Internal and external carcass lengths, chest width and depth, and rump width and depth also did not differ (P>0.05) (Table 4). Rump width showed an increasing (P=0.088) trend when soybean meal was replaced by cottonseed cake.

The pH measured immediately after slaughter, which averaged 6.55, color, measured in its coordinates $L^{*}$ (37.40), $a^{*}$ (19.49), and $b^{*}$ (5.42), CL (25.87 g/kg), and SF (18.88 N/cm) in the longissimus lumborum muscle were not affected by the dietary replacement of soybean meal with cottonseed cake (P > 0.05) (Table 5).

**Table 4:** Morphometric measurements (cm) of the carcass of feedlot goats fed diets containing cottonseed cake replacing soybean meal.

Item	Cottonseed cake level				SEM${}^{*}$	P value	
	(g/kg per dry matter)						
	0	330	660	1000		Linear	Quadratic
External length	42.00	40.87	42.25	42.50	2.81	0.534	0.547
Internal length	50.42	50.50	49.00	49.37	2.21	0.235	0.835
Chest width	15.00	14.37	16.25	15.43	1.94	0.345	0.863
Rump width	11.71	12.81	13.43	13.50	1.89	0.088	0.477
Chest depth	23.57	23.93	24.43	26.00	3.43	0.198	0.659
Rump circumference	42.85	42.75	42.93	38.12	6.51	0.214	0.355
Thigh circumference	31.78	32.62	32.25	30.37	2.61	0.290	0.191
Leg length	41.14	41.62	40.62	39.75	2.20	0.173	0.442

${}^{*}$ Standard error of the mean.

**Table 5:** Qualitative traits of the longissimus lumborum muscle of feedlot goats fed diets containing cottonseed cake replacing soybean meal.

Item	Cottonseed cake level				SEM${}^{1}$	P value	
	(g/kg per dry matter)						
	0	330	660	1000		Linear	Quadratic
pH	6.46	6.71	6.51	6.53	0.28	0.500	0.959
Color${}^{2}$	–	–	–	–	–	–	–
$L^{*}$	37.62	36.21	37.15	38.61	2.28	0.303	0.114
$a^{*}$	19.39	19.67	19.98	19.39	1.61	0.180	0.290
$b^{*}$	6.02	5.15	5.39	5.11	1.11	0.225	0.455
Cooking losses, g/kg	261.2	224.6	292.4	256.6	62.2	0.282	0.059
Shear force, N/cm	19.32	17.55	18.83	19.81	0.43	0.157	0.656

${}^{1}$ Standard error of the mean. ${}^{2}$ $L^{*}$: lightness. $a^{*}$: redness. $b^{*}$: yellowness.

The experimental diets did not alter (P > 0.05) the moisture (746.5 g/kg), protein (210.2 g/kg), or total lipid (33.2 g/kg) contents of goat meat. By contrast, the MM content decreased (from 10.8 to 9.7 g/kg; P=0.001) and the cholesterol levels increased (from 50.85 to 70.55 mg/100 g of meat; P=0.006) as soybean meal was replaced with cottonseed cake in the diets (Table 6).

Cooking losses (P=0.059), moisture (P=0.084), and total lipids (P=0.080) of the goat meat showed quadratic effects when soybean meal was replaced by cottonseed cake.

**Table 6:** Centesimal composition (g/kg) and cholesterol content (mg/100 g of meat) of the longissimus lumborum muscle of feedlot goats fed diets containing cottonseed cake replacing soybean meal.

Item	Cottonseed cake level				SEM${}^{1}$	P value	
	(g/kg per dry matter)						
	0	330	660	1000		Linear	Quadratic
Moisture	741.0	747.1	750.1	747.6	15.8	0.459	0.084
Mineral matter${}^{2}$	10.8	10.7	9.3	9.7	0.6	0.001	0.277
Protein	214.9	208.1	207.6	210.3	7.8	0.202	0.325
Total lipids	33.3	34.1	33.0	32.4	7.8	0.095	0.060
Cholesterol${}^{3}$	50.85	61.83	66.48	70.55	12.12	0.006	0.564

${}^{1}$ Standard error of the mean. ${}^{2}$ Y=10.825-0.0014x, $R^{2}=0.67$. ${}^{3}$ Y=52.917+0.0192x, $R^{2}=0.93$.

## 4 Discussion

The similar intake values of the nutritional components indicate that the nutritional potential of cottonseed was effective in replacing soybean meal in goat diets. Therefore, in the current study, the cottonseed inclusion in the diet did not promote satiety effects related to the chemical effect of meeting the energy requirements or the physical effect of rumen distension due to fiber intake (Allen, 2014; R. V. M. M. Silva et al., 2016). The quadratic effect of EE intake is related to the EE content of the diets, probably at 44.3 g/kg per dry matter, the EE content of the diet when substituting soybean meal for cottonseed cake is low, thus promoting a lower intake of this nutrient. This theory can be supported by the trend observed in the intake of NFC, which decreases as the content of NFC in the diet is reduced.

On the other hand, considering the DM intake at the highest level of replacement of soybeans with cottonseed cake, EE intake was lower (51.0 %) and CP intake was higher (42.9 %) than expected. Considering the higher EE content and lower CP of cottonseed cake (7.6 % and 24.0 %, respectively) compared to soybean meal (1.9 % and 39.0 %, respectively) (R. V. M. M. Silva et al., 2016), the results of the current study indicate that there was a possible certain degree of feeding selectivity. The behavior of selectivity can be corroborated with the results found by Castro et al. (2020). These authors who evaluated cottonseed outlined in diets for finished sheep concluded that changes in nutrient intake were also due to selectivity. Furthermore, it is important to bear in mind that goats are the animals with the highest selectivity compared to sheep and cattle (Mohammed et al., 2020).

The similar values on quantitative traits and cut yields of the goats are probably related to the uniformity of age, initial live weight, breed, and sex class as well as the similar intakes of DM across the treatment groups. Therefore, the nutritional changes due to the total substitution of soybean meal for cottonseed cake did not promote positive or negative changes in the productive traits of the goats. These results were also observed in lambs fed with the same levels of substitution of soybean meal for cottonseed cake (R. V. M. M. Silva et al., 2016).

Hot carcass yield and LEA were higher than the values described in many other studies with goats (Silva et al., 2011; Browning et al., 2012; Yusuf et al., 2014). This fact suggests that the animals evaluated in the present study had a good genetic constitution and were properly managed and fed.

The results obtained for LEA and BFT can be attributed to the fact that the goats were slaughtered at similar body weights, which corroborates the following inference drawn by Osório (2002): in carcasses of similar weights and fatness degrees, practically all body regions will have similar proportions, irrespective of the breed. In this study, this assertion was confirmed by the yield of primal cuts and the carcass morphometric measurements (Tables 3 and 4).

Loin-eye area and BFT are traits used as indicators of muscularity and degree of fatness in the carcass, respectively (Matarim, 2015). The carcass cuts, in turn, depend on their appearance to ensure a more effective utilization of the meat, allowing for increased selling prices (FAO, 1991).

Although no changes were detected in the carcass biometric measurements, these assessments are important in a production system, considering that such measurements are correlated with in vivo measurements and can be used along with them or separately (Bautista-Díaz et al., 2017).

The higher pH value of goat meat is probably related to species rather than diet, considering that the pH of goat meat is higher (Shija et al., 2013). The average meat pH value (6.55) recorded in this study was close to the 6.55 observed by Shija et al. (2013) and to the 6.53 reported by Pophiwa et al. (2017) in goat meat immediately after slaughter. Those authors also observed a decline curve of natural pH until its stabilization, between 5.4 and 5.8, which is considered normal for this species (Puolanne, 2017). The meat of the animals evaluated in the current study behaved likewise, suggesting normal rigor mortis development and inexistence of pre-slaughter stress (Della Malva et al., 2016; Pereira et al., 2016; T. M. Silva et al., 2016). Therefore, meat quality would not be compromised (Gallo et al., 2018).

Results for meat color, CL, and SF (Table 5) were similar to those reported by Ribeiro et al. (2018), who examined the meat quality of feedlot Boer crossbred goats fed diets that also contained biodiesel by-products. Meat color is the basic qualitative attribute that most influences the consumer's choice, eliciting their interest in consuming or rejecting the product (Suman and Joseph, 2012). Color is an attribute affected by the physical and chemical characteristics of meat, such as pH, marbling, thickness of subcutaneous fat, and lipid and moisture content (Webb, 2014). Therefore, the lack of effect of cottonseed cake on the color of the meat may be correlated to the lack of effect of this on the physical and chemical values of the meat.

Cooking loss, in turn, is an important parameter in the evaluation of meat quality, as it is associated with the yield of meat during preparation for consumption, besides influencing its juiciness and tenderness (Campos et al., 2017). Lastly, texture is the most important factor in determining meat quality from the consumer's perspective, since it is related to how easily the meat is chewed as well as to sensations perceived by the consumer upon chewing (Mennecke et al., 2007; Liu et al., 2017). These three attributes are among those deemed most important at the time of purchase of meat (Madruga et al., 2008; Girard et al., 2016; Muela et al., 2016).

The centesimal composition of the longissimus lumborum of the goats evaluated in this study was close to values described in the literature for goat meat (Kessler et al., 2014; T. M. Silva et al., 2016; Ribeiro et al., 2018). The centesimal composition of meat is important because it basically expresses its nutritional value as well as the proportion of each nutrient in the diet.

Although the cholesterol contents in the goats' meat increased with the dietary replacement, the observed values were lower than the 75 mg/100 g of goat meat recommended by the U.S. Department of Agriculture (USDA, 2011). They agree, however, with the 64.48 and 69.89 mg/100 g of meat found by Madruga et al. (2005) in Boer crossbred and unidentified-breed goats, respectively, 63.92 mg/100 g of meat in native goats reported by Bonvillani et al. (2010), and 74.76 mg/100 g of meat in Angora crossbred goats described by Kessler et al. (2014). Based on the cholesterol values found in the goats' meat in the present study, it is possible to state that their meat can make up a healthy diet, considering that the World Health Organization recommends a daily cholesterol intake of less than 300 mg or 200 mg for people with a history of heart disease.

## 5 Conclusions

Replacing soybean meal with cottonseed cake in feedlot goat diets does not compromise the carcass traits or meat quality of those animals. We recommend using up to 100 % of cottonseed cake as the protein source in feedlot goat diets.

## Data Availability

The original data used in this study are available from the corresponding author upon request.
